# Unveiling Immunomodulatory Effects of *Euglena gracilis* in Immunosuppressed Mice: Transcriptome and Pathway Analysis

**DOI:** 10.4014/jmb.2401.01006

**Published:** 2024-02-19

**Authors:** Seon Ha Jo, Kyeong Ah Jo, Soo-yeon Park, Ji Yeon Kim

**Affiliations:** 1 Department of Food Science and Biotechnology, Seoul National University of Science and Technology, Seoul 01811, Republic of Korea; 2 Department of Nano Bio Engineering, Seoul National University of Science and Technology, Seoul 01811, Republic of Korea

**Keywords:** Cyclophosphamide, *Euglena*, beta-glucan, immunomodulation, transcriptome

## Abstract

The immunomodulatory effects of *Euglena gracilis* (*Euglena*) and its bioactive component, β-1,3-glucan (paramylon), have been clarified through various studies. However, the detailed mechanisms of the immune regulation remain to be elucidated. This study was designed not only to investigate the immunomodulatory effects but also to determine the genetic mechanisms of *Euglena* and β-glucan in cyclophosphamide (CCP)-induced immunosuppressed mice. The animals were orally administered saline, *Euglena* (800 mg/kg B.W.) or β-glucan (400 mg/kg B.W.) for 19 days, and CCP (80 mg/kg B.W.) was subsequently administered to induce immunosuppression in the mice. The mice exhibited significant decreases in body weight, organ weight, and the spleen index. However, there were significant improvements in the spleen weight and the spleen index in CCP-induced mice after the oral administration of *Euglena* and β-glucan. Transcriptome analysis of the splenocytes revealed immune-related differentially expressed genes (DEGs) regulated in the *Euglena*- and β-glucantreated groups. Gene ontology (GO) and Kyoto Encyclopedia of Genes and Genomes (KEGG) analyses indicated that pathways related with interleukin (IL)-17 and cAMP play significant roles in regulating T cells, B cells, and inflammatory cytokines. Additionally, Ptgs2, a major inflammatory factor, was exclusively expressed in the *Euglena*-treated group, suggesting that *Euglena*’s beneficial components, such as carotenoids, could regulate these genes by influencing immune lymphocytes and inflammatory cytokines in CCP-induced mice. This study validated the immunomodulatory effects of *Euglena* and highlighted its underlying mechanisms, suggesting a positive contribution to the determination of phenotypes associated with immune-related diseases and the research and development of immunotherapies.

## Introduction

*Euglena gracilis*, commonly referred to as *Euglena*, stands out as a microalgae that is rich in a variety of beneficial nutrients, such as vitamins, minerals, carotenoids, and chlorophyll [1]. Possessing attributes of both a plant and an animal, *Euglena* is a promising biological resource frequently employed as a food or health supplement [2]. Additionally, *Euglena* species accumulate a storage polysaccharide in their body characterized by linear β-1,3-glucan chains [3, 4]. In contrast to the β-glucans in the cell walls of plants and bacteria, the β-glucan in *Euglena* is stored in granules called paramylon in the cytoplasm, constituting up to 90% of *Euglena* [5]. *Euglena* and β-glucan have been shown to improve health by enhancing immunity, inhibiting atopic dermatitis, and modulating gut microbial composition [6, 7]. In vitro, in vivo, and clinical trial studies have demonstrated that *Euglena* and β-glucan activate the immune system by releasing cytokines and inducing the immune cells such as natural killer cells or T lymphocytes [8-10]. They also activate the production of TNF-alpha in macrophages and modulate the expression of various cytokines [10]. Although they are known for their antitumor, anti-inflammatory, and antiallergic effects, the detailed genetic immune mechanisms involved remain to be elucidated. The human immune system protects the body from pathogens and cancer cells, providing defense through immune cells and antibodies against infections and diseases [11]. Serving as the primary barrier, the innate immune system initiates immune responses through collaborative interactions among macrophages, lymphocytes, and their products, such as cytokines [12]. Immune cells form a complex network to respond to pathogens, eliminate abnormal cells, and ultimately strengthen immune defenses across the nuclear factor (NF)-kB and mitogen-activated protein kinase (MAPK) signaling pathways, primarily in the spleen and lymph nodes [13, 14]. Disruptions in immune balance and immune deficiency may lead to conditions such as atopic disease and intestinal disorders [15, 16]. Research indicates that many diseases and complications are epidemiologically associated with immune suppression [17]. Cyclophosphamide (CCP) is a cytotoxic alkylating agent, that is widely used as an anticancer agent for a variety of diseases [18]. CCP induces cytotoxicity in proliferating lymphocytes, demonstrating immunosuppressive effects [19]. Thus, it was used to establish an experimental model applicable to the evaluation of immunomodulation by antibiotics in a previous study [20]. Advancements in DNA sequencing technologies, particularly next-generation sequencing (NGS), offer powerful tools for analyzing diverse gene expression patterns and identifying differentially expressed genes (DEGs) [21]. NGS has enabled scientific achievements and biological applications that were previously inconceivable. Recent research utilizing NGS for comprehensive transcriptome analyses of diverse organisms has demonstrated promising prospects for immunotherapy for immune-related disorders [22, 23]. Moreover, NGS can provide significant insights into the immunological characteristics of organisms by examining the impact of transcriptional profiles on the immune response [24]. Advanced RNA sequencing (RNA-seq) technology, an NGS method, can be used to interpret the genetic aspects of immune regulation and identify previously unknown immunological points. Despite previous studies confirming the immunomodulatory effects of *Euglena*, the underlying cellular and genetic mechanisms have not been elucidated. Therefore, the primary objective of this study was to validate the underlying immunomodulatory mechanisms through comprehensive genetic analysis, identifying specific genes and pathways selectively expressed in response to *Euglena* and β-glucan. In the experimental section, *Euglena* and β-glucan were administered to CCP-induced immunosuppressed murine models to explore their immune-enhancing effects. After the identification of the DEGs in the mouse model through RNA sequencing, the network between pathways and genes was visualized in various formats, providing a systematic overview. This procedure was subsequently validated to confirm significantly enhanced pathways and genes.

## Materials and Methods

### Materials

High-glucose Dulbecco’s modified Eagle’s medium (DMEM), Dulbecco’s phosphate-buffered saline (DPBS), fetal bovine serum (FBS), 1 M hydroxyethyl piperazine ethane sulfonic acid (HEPES) buffer, and penicillin–streptomycin mixtures were obtained from Biowest (France). Roswell Park Memorial Institute (RPMI)-1640 medium was obtained from Welegene (Korea). CCP, red blood cell (RBC) lysis buffer, amphotericin B, and trypan blue were obtained from Sigma–Aldrich (USA). The YAC-1 cell line, utilized for measuring NK cell activity, was obtained from the Korean Cell Line Bank (Republic of Korea).

### Sample Preparation: *Euglena* and β-glucan Powder

Euglena and β-glucan powder were obtained from Daesang Corp. R&D Center (Republic of Korea). *Euglena gracilis* DSW1 cells (KCTC 13930BP) were cultured for 6 days in medium supplemented with L-glutamic acid, DL-malic acid, glucose, dibasic potassium phosphate, and cyanocobalamin. Following the 6-day incubation period in a jar fermenter, the *Euglena* cells were subjected to centrifugation at 4,000 ×*g* for 10 min and then washed with distilled water. The collected cells were sterilized and dried. β-glucan powder was prepared by centrifuging *Euglena* cells at 4,000 ×*g* for 10 min. After the pH was adjusted to 12.5 with NaOH, the cells were extracted at 60°C for 1 h. Subsequent centrifugation under the same conditions was performed, followed by sterilization and drying. The resulting paramylon exhibited a β-glucan content of 97%. Analysis of the β-glucans was conducted using 1H NMR spectroscopy at the Korea Basic Science Institute (Republic of Korea), confirming their identity as β-1,3-glucans. The prepared *Euglena* powder contained 665.61 mg/g of β-glucans. Based on this, the concentration of the β-glucan sample administered to the animals was set at half the concentration of the *Euglena* sample. Thus, the concentrations of the *Euglena* and β-glucan samples were set at 800 mg/kg B.W. and 400 mg/kg B.W., respectively.

### Animal Study Design

Five-week-old male ICR mice were procured from Hana Biotech (Republic of Korea) for this study. The mice were housed under a 12 h light/dark cycle with unrestricted access to food and water. Following a one-week acclimation period at the Dongnam Chemical Research Institutés animal facility (Animal Facility Registration No. 412, Republic of Korea), the mice were grouped as follows (13 mice per group): normal control (CON), 80 mg/kg body weight (B.W.) cyclophosphamide-only (CCP), CCP + 800 mg/kg B.W. *Euglena* (E800), and CCP + 400 mg/kg B.W. β-glucan (B400). The CON and CCP groups received oral saline, while the E800 and B400 groups (sample groups) were orally administered *Euglena* and β-glucan, respectively, for 19 days. The E800 and B400 groups also received daily intraperitoneal injections of CCP diluted in saline for 3 days to induce immunosuppression. This immunosuppressive treatment started on the 17th day of test substance administration. On the 19th day, the experimental animals were euthanized with CO_2_, followed by laparotomy. Blood samples were collected from the abdominal aorta using a 1 ml syringe. The spleen, Peyer's patches, and mesenteric lymph nodes were harvested and weighed. Additional specimens were preserved in RPMI 1640 medium for further analysis. Changes in body weight were calculated using the following formula: change in body weight (BW) (%) = BW (g) on Day 19/BW (g) on Day 17 × 100. This research was conducted in accordance with the policies and regulations of the Institutional Animal Care and Use Committee (IACUC) of Dongnam Chemical Research Institute (Approval No. SEMI-23-007).

### Sample Preparation for RNA Sequencing Analysis

The purity of the RNA (*n* = 5 per group) was assessed by analyzing 1 μl of total RNA extracted on a NanoDrop8000 spectrophotometer. The integrity of the total RNA was verified using an Agilent Technologies 2100 Bioanalyzer (Agilent, USA), which provided an RNA integrity number (RIN) value. mRNA sequencing libraries were generated following the manufacturer's instructions for the TruSeq stranded mRNA library prep kit (Illumina, USA). To isolate and fragment mRNA from total RNA, poly-T-oligo-attached magnetic beads were used for two rounds of purification. Cleaved RNA fragments primed with random hexamers were reverse transcribed into first-strand cDNA using reverse transcriptase, random primers, and dUTP in place of dTTP. The resulting products were purified and enriched through PCR to create the final strand-specific cDNA library. The quality of the amplified libraries was confirmed through automated electrophoresis (Agilent). Following quantitative polymerase chain reaction (qPCR) using KAPA SYBR FAST qPCR Master Mix (Kapa Biosystems, USA), index-tagged libraries were combined in equimolar amounts for pooling. RNA sequencing was conducted on a NovaSeq 6000 system (Illumina) in accordance with the manufacturer’s protocols.

### Identification of DEGs

Reads for each sample were mapped to a reference (*Mus musculus* GRCm39) by Kallisto v0.46.1 (USA) [25]. The alignment results were subsequently added to the edgeR package to determine the DEGs. The DEG analysis was conducted with two different comparison designs: between the CON and the E800 groups (CON vs. E800) and between the CON and the B400 groups (CON vs. B400). Significant DEGs were selected based on the criteria of a false discovery rate (FDR) < 0.05 and |log2 Fold Change| ≥ 1.

### Pathway Enrichment Analysis

For a deeper understanding of the biological processes involved and the gene pathways enriched, functional enrichment analysis of DEGs was conducted utilizing the Database for Annotation, Visualization, and Integrated Discovery (DAVID) (http://david.abcc.ncifcrf.gov/) [26]. Gene Ontology (GO) enrichment terms and Kyoto Encyclopedia of Genes and Genomes (KEGG) pathway enrichment data were considered significant at thresholds of *p* < 0.05. Additionally, a protein‒protein interaction (PPI) network was established using the online Search Tool for the Retrieval of Interacting Genes (STRING) (https://string-db.org/), aiming to identify possible relationships [27]. In the constructed network, nodes and connecting edges symbolize biological molecules and their relationships, respectively. The DEGs and pathways were visually represented using Cytoscape (https://cytoscape.org/) for network visualization [28].

### Quantitative Reverse Transcription Polymerase Chain Reaction (RT–qPCR)

Total RNA was isolated from the splenocytes using TRIzol reagent (Life Technologies, USA). Subsequently, cDNA was synthesized with a cDNA reverse transcription kit (Roche, Switzerland). RT‒qPCR was carried out with specific primers for interferon-γ (IFN-γ), interleukin (IL)-17, Ptgs2, IL-2, and glyceraldehyde 3-phosphate dehydrogenase (GAPDH), as shown in Table 1. The gene expression levels of IFN-γ, IL-17, Ptgs2, and IL-2 were quantified using a Light Cycler 96 system (Hoffmann La Roche) based on the Universal Probe Library (UPL) method. The relative mRNA expression, normalized to that of GAPDH, was calculated using the comparative CT method.

### Statistical Analysis

All the results are expressed as the mean ± standard error of the mean (SEM). Statistical significance was assessed using one-way ANOVA followed by Duncan’s multiple range test (SAS 9.4, SAS Institute, USA). Different letters indicate significant differences between the groups (*p* < 0.05).

## Results

### Effects of *Euglena* on Body Weight in a CCP-Induced Mouse Model

**Fig. 1:**
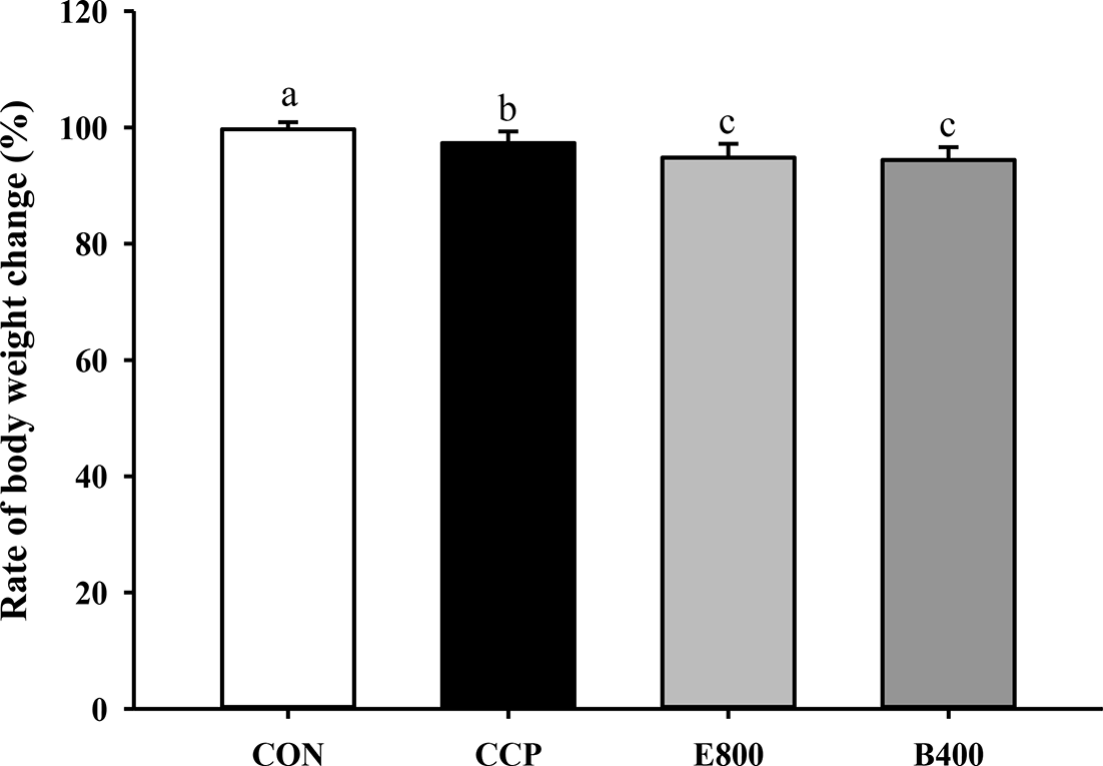
Rate of body weight change after treatment with cyclophosphamide. CON, normal control; CCP, cyclophosphamide (80 mg/kg B.W.); E800, *Euglena* (800 mg/kg B.W.) and cyclophosphamide (80 mg/kg B.W.). B400, β-glucan (400 mg/kg B.W.) and cyclophosphamide (80 mg/kg B.W.) (*n* = 13 for each group). Rate of body weight change after CCP injection. The data represent the mean ± SEM. Different letters indicate significant differences (*p* < 0.05) according to Duncan’s multiple range test.

To investigate the immunosuppressive effects of CCP on a mouse model, changes in B.W. were measured on Day 19 compared to Day 17, the first day of CCP administration. As shown in Fig. 1, the percentage of B.W. change (%) was significantly lower in the CCP-induced groups (CCP, E800, and B400) than in the CON group (*p* < 0.0001).

### Effects of *Euglena* on Organ Weight in a CCP-Induced Mouse Model

Critical immune-related organs, such as the spleen, Peyer’s patches, and mesenteric lymph nodes (MLNs), were measured to explore the immune-enhancing effects of *Euglena* and β-glucan on immunosuppressed mice (Table 2). The weights of the spleen, Peyer’s patches, and MLN were significantly lower in the CCP-induced groups than in the CON groups (*p* < 0.0001, consistent across all three analyzed organs). Among these groups, the spleen weights of the E800 and B400 groups were significantly greater than that of the CCP group. However, there were no significant differences in Peyer’s patch or MLN weights between the CCP, E800, and B400 groups.

### Effects of *Euglena* on the Spleen Index of a CCP-Induced Mouse Model

**Fig. 2:**
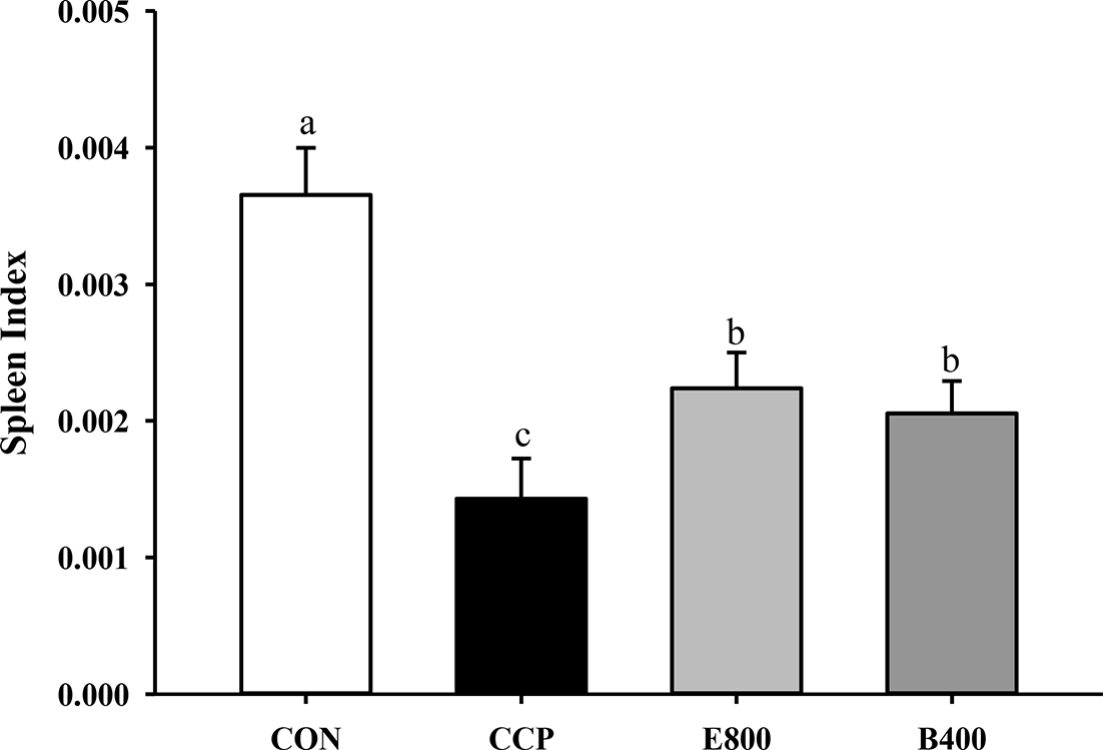
Effects of *Euglena* and β-glucan on the spleen index. CON, normal control; CCP, cyclophosphamide (80 mg/kg B.W.); E800, *Euglena* (800 mg/kg B.W.) and cyclophosphamide (80 mg/kg B.W.); B400, β-glucan (400 mg/kg B.W.) and cyclophosphamide (80 mg/kg B.W.) (*n* = 13 for each group). The spleens of all the mice were weighed, and the spleen indices were calculated. The data represent the mean ± SEM. Different letters indicate significant differences (*p* < 0.05) according to Duncan’s multiple range test.

As illustrated in Fig. 2, the spleen indices were significantly lower in the CCP group than in the CON group (*p* < 0.0001). In the CCP-induced groups, the spleen indices were significantly greater than those in the CCP group, with approximately 1.56-fold and 1.43-fold increases in the E800 and B400 groups, respectively.

### Identification of DEGs

**Fig. 3:**
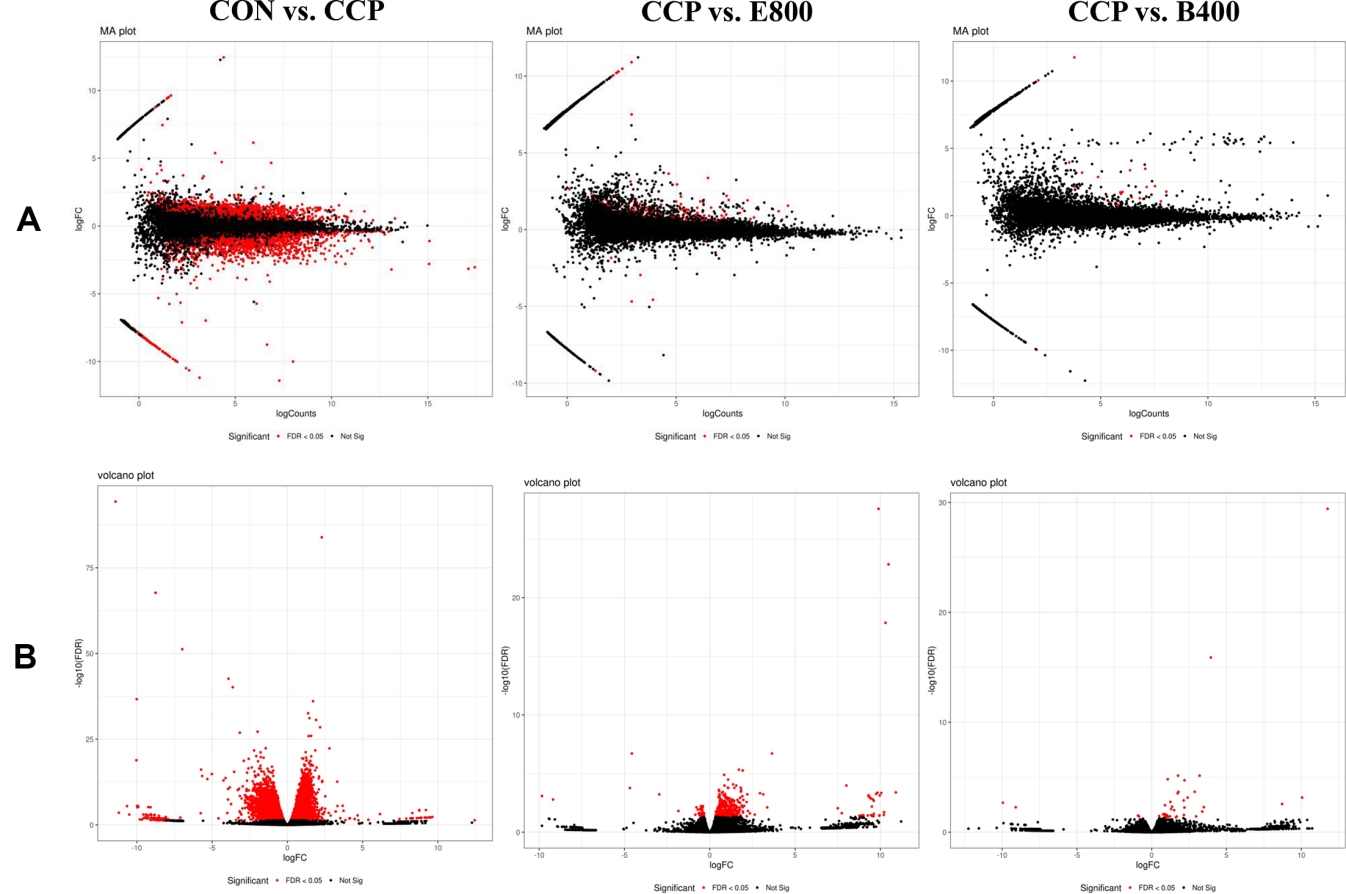
MA and volcano plot of DEGs after treatment with *Euglena* and β-glucan. CON, normal control; CCP, cyclophosphamide (80 mg/kg B.W.); E800, *Euglena* (800 mg/kg B.W.) and cyclophosphamide (80 mg/kg B.W.); B400, β-glucan (400 mg/kg B.W.) and cyclophosphamide (80 mg/kg B.W.) (*n* = 5, randomly selected mice for each group). Differentially expressed genes with an FDR < 0.05 are marked in red.

**Fig. 4:**
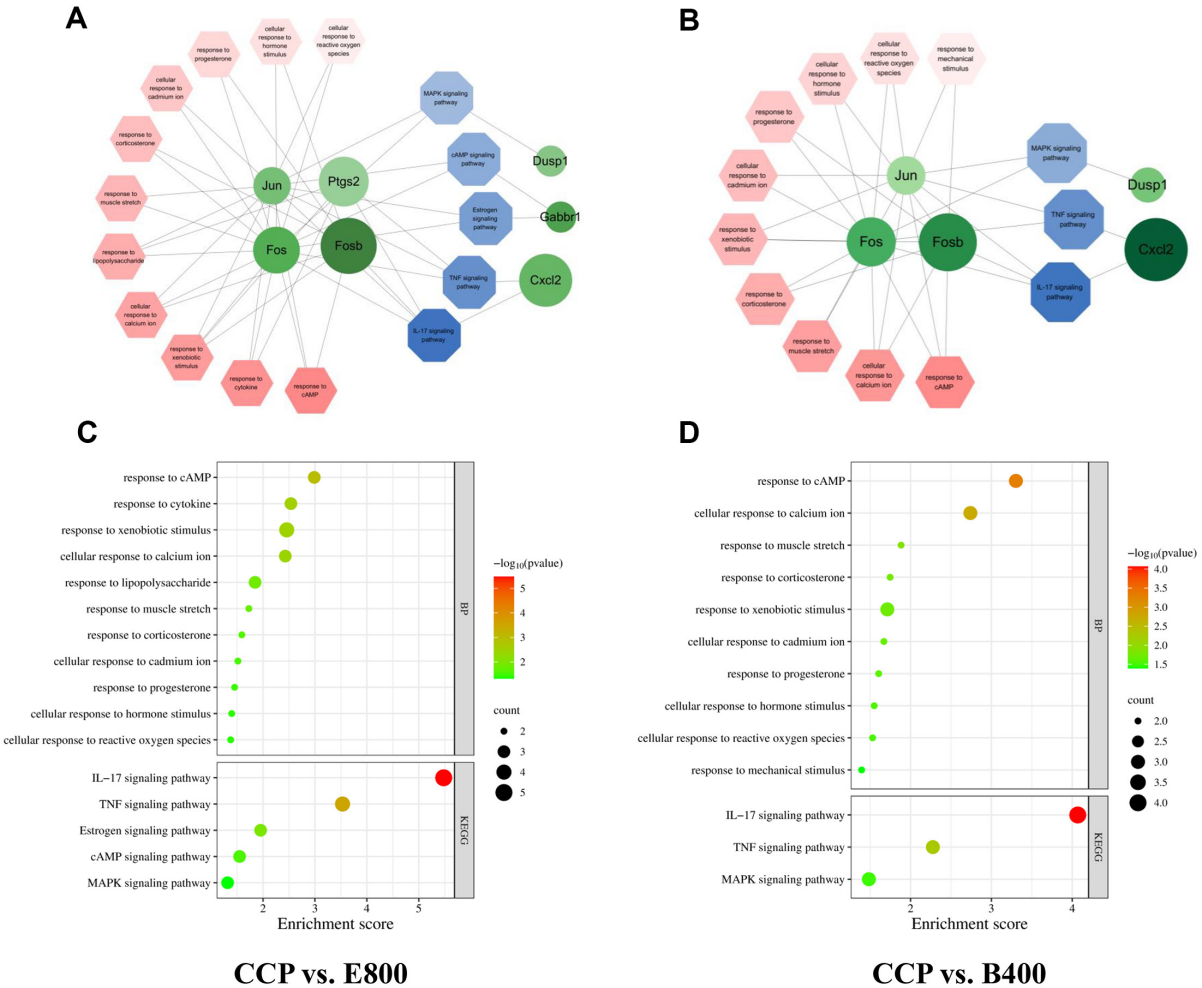
Visualization of immune-related pathways and genes in CCP-induced immunosuppressed mice after treatment with *Euglena* and β-glucan. CON, normal control; CCP, cyclophosphamide (80 mg/kg B.W.); E800, *Euglena* (800 mg/kg B.W.) and cyclophosphamide (80 mg/kg B.W.). B400, β-glucan (400 mg/kg B.W.) and cyclophosphamide (80 mg/kg B.W.) (*n* = 5, randomly selected mice for each group). (**A**) Network visualization of immune-related pathways and genes in the CCP vs. E800 comparison. (**B**) Network visualization of immune-related pathways and genes in the CCP vs. B400 comparison. The colors of the shapes (red for GO terms, blue for KEGG pathways, and green for genes) reflect the corresponding entities. The brightness of a node in the network reflects the *p* value, and the size of the node indicates the log (fold change) value of gene expression. (**C**) Comparison of immune-related pathways and genes between the CCP and E800 cohorts using a bubble chart visualization. (**D**) Comparison of immune-related pathways and genes between CCP and B400 using a bubble chart visualization. The color of the bubble and enrichment score denote the –log (*p* value), and the size of the bubble denotes the gene count.

Based on the FDR < 0.05 and |log2 Fold Change|≥ 1 criteria, a total of 2312, 167, and 32 DEGs were identified in the three DEG sets of immunosuppressed mice treated with *Euglena* and β-glucan: CON vs. CCP, CCP vs. E800 and CCP vs. B400, respectively (Table 3). Within the CCP vs. E800 comparison, 167 DEGs were observed, comprising 161 upregulated genes and 6 downregulated genes. In the CCP vs. B400 comparison, 32 DEGs were detected, including 30 upregulated genes and 2 downregulated genes. GO analysis revealed 1922, 30, and 15 DEGs in the CON vs. CCP, CCP vs. E800, and CCP vs. B400 comparisons, respectively. Moreover, KEGG analysis revealed 905, 12, and 10 DEGs in the CON vs. CCP, CCP vs. E800, and CCP vs. B400 comparisons, respectively. The DEGs associated with all the upregulated and downregulated genes were visualized with different plots, such as MA plots and volcano plots (Figs. 3 and 4), which clearly illustrated the distribution of genes whose expression significantly changed. Notably, the CON vs. CCP comparison exhibited the highest number of DEGs, with the CCP vs. E800 comparison showing more DEGs than the CCP vs. B400 comparison.

### Immune-Related GO and KEGG Analyses of DEGs

To clarify the mechanism of the immune response to *Euglena* and β-glucan, immune-related pathways were selected from the GO and KEGG analyses based on the QuickGO database about immune responses [29]. The outcomes of the analysis of DEGs are summarized in Table 4, which shows the numbers of immune-related pathways and genes. According to the KEGG analysis, five pathways were identified in the CCP vs. E800 comparison, and three were identified in the CCP vs. B400 comparison. The sets of DEGs associated with *Euglena* and β-glucan treatment were analyzed, and 7 and 5 genes, respectively, were identified. GO_BP analysis revealed 11 immune-related biological process terms in the CCP vs. E800 comparison and 10 out of 18 in the CCP vs. B400 comparison. Additionally, 5 and 3 immune-related genes were identified in the *Euglena*- and β-glucan-treated DEG sets, respectively.

As indicated in Table 5, according to KEGG analysis, the IL-17 signaling pathway exhibited the most significant enrichment (*p* = 3.32E-06) and the highest number of annotated DEGs in the CCP vs. E800 comparison. Other notable immune-related pathways included the tumor necrosis factor (TNF), estrogen, cAMP, and MAPK signaling pathways. All the KEGG results for the CCP vs. E800 comparison, except for the estrogen-related pathway, were consistent with those for the CCP vs. B400 comparison (Table 6).

The GO term “response to cAMP” showed the most significant enrichment in both the CCP vs. E800 and CCP vs. B400 comparisons (*p* = 1.03E-03, 4.98E-04), with annotated genes such as Jun, Fos, and Fosb. The GO terms included “response to cytokine” and “response to lipopolysaccharide” only for the CCP vs. E400 comparison, and “cellular response to reactive oxygen species” was included for both the CCP vs. E800 and CCP vs. B400 comparisons.

The DEGs identified from the KEGG analysis of the CCP vs. E800 comparison revealed a total of seven upregulated genes, namely, Fosb, Fos, Ptgs2, Jun, Cxcl2, Gabbr1, and Dusp1. The results from the GO_BP analysis demonstrated enrichment of DEGs such as Fosb, Fos, Ptgs2, and Jun. In the CCP vs. B400 comparison, 5 upregulated genes (Fosb, Fos, Jun, Cxcl2, and Dusp1) were identified via KEGG analysis, and 3 upregulated genes (Fosb, Fos, and Jun) were identified via GO_BP related to the immune system. The analyzed immune-related DEGs were consistent between the CCP vs. E800 and CCP vs. B400 comparisons, except for Ptgs2 and Gabbr1.

### Visualization of the Immune-Related Network between Pathways and Genes

Fig. 4A and 4B illustrate immune-related DEGs following the ingestion of *Euglena* and β-glucan, along with the analysis of KEGG pathways and GO_BP terms. Notably, Fosb, Ptgs2, and Cxcl2 exhibited relatively high fold changes in expression in the CCP vs. E800 comparison. Gabbr1 and Fosb exhibited significantly pronounced differences in expression compared to the other genes with high *p* values in the same DEG set. Additionally, compared with those of the other genes, the enrichment of Fos, Fosb, Jun, and Ptgs2 in the CCP vs. E800 comparison showed intricate associations with various immune-related pathways and terms. Both Fosb and Cxcl2 exhibited significant changes in gene expression in response to β-glucan. Cxcl2 and Fosb exhibited significantly higher *p*-values in the CCP vs. B400 comparison. Additionally, the pathway with the highest gene count and *p* value in both the CCP vs. E800 and CCP vs. B400 comparisons was the IL-17 signaling pathway. Moreover, the GO_BP terms with the highest gene count and *p* value were associated with the cAMP signaling pathway (Fig. 4C and 4D).

### Biological Interpretation via PPI Network Analysis

**Fig. 5:**
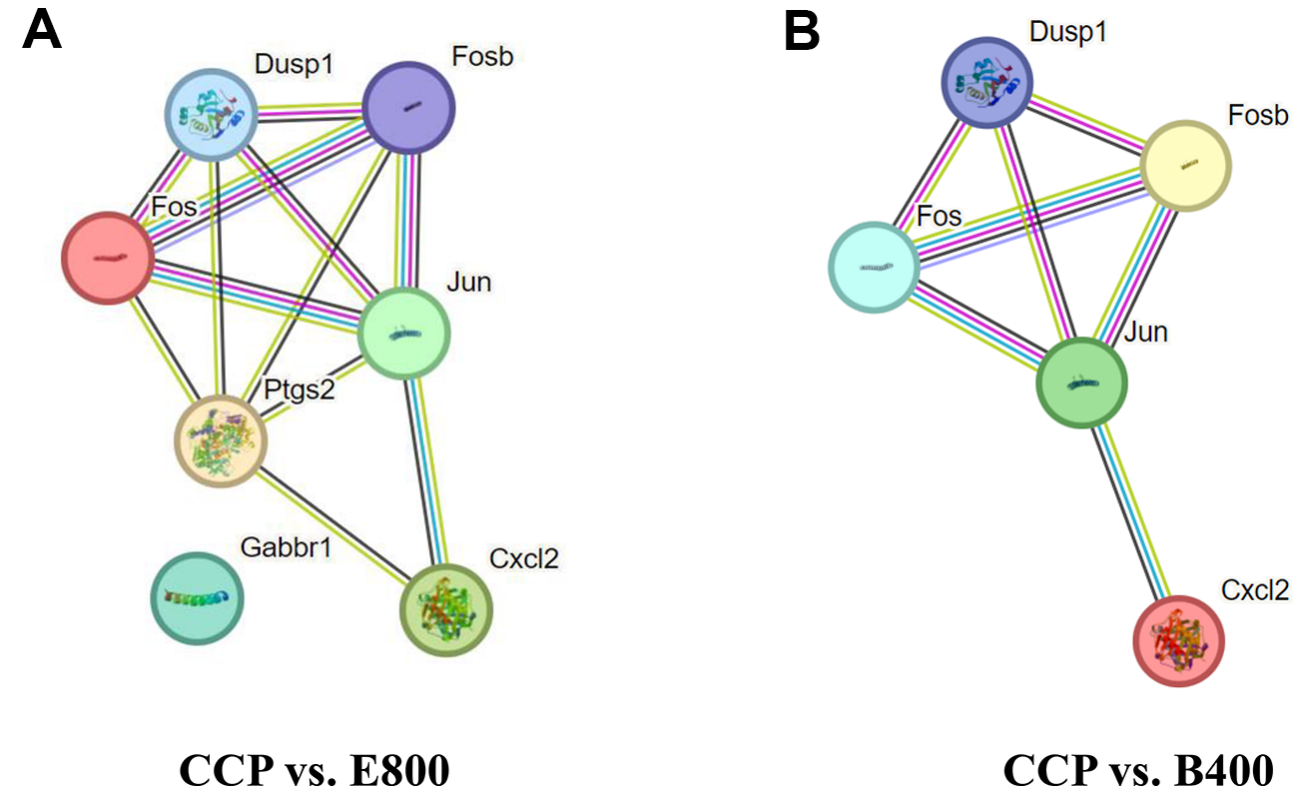
Protein‒protein interaction (PPI) network for immune-related genes in CCP-induced immunosuppressed mice after treatment with *Euglena* and β-glucan. CON, normal control; CCP, cyclophosphamide (80 mg/kg B.W.); E800, *Euglena* (800mg/kg B.W.) and cyclophosphamide (80mg/kg B.W.). B400, β-glucan (400 mg/kg B.W.) and cyclophosphamide (80 mg/kg B.W.) (*n* = 5, randomly selected mice for each group). (**A**) Gene network of a total of 7 immune-related genes in the CCP vs. E800 comparison. (**B**) Gene network of a total of 5 immune-related genes in the CCP vs. B400 comparison. The network nodes represent the genes, and the edges represent gene–gene associations.

To unravel the interactions among DEGs, a PPI network was constructed for the seven selected immune-related DEGs (Fosb, Fos, Jun, Cxcl2, Dusp1, Ptgs2, and Gabbr1). All the analyzed immune-related DEGs were matched with proteins in the STRING database (species: *Mus musculus*). The constructed PPI network for the CCP vs. B400 comparison contained 5 nodes and 7 edges, while that for the CCP vs. E800 comparison contained 7 nodes and 12 edges (Fig. 5). Fosb, Fos, and Jun exhibited the most intimate relationships in both the CCP vs. E800 and CCP vs. B400 comparisons. Fosb, Fos, Jun, Cxcl2, and Dusp1 were annotated in both DEG sets, while Ptgs2 and Gabbr1 were enriched only in the CCP vs. E800 comparison. Ptgs2 was more closely related to other genes than was Gabbr1.

### Validation of Gene Expression

**Fig. 6:**
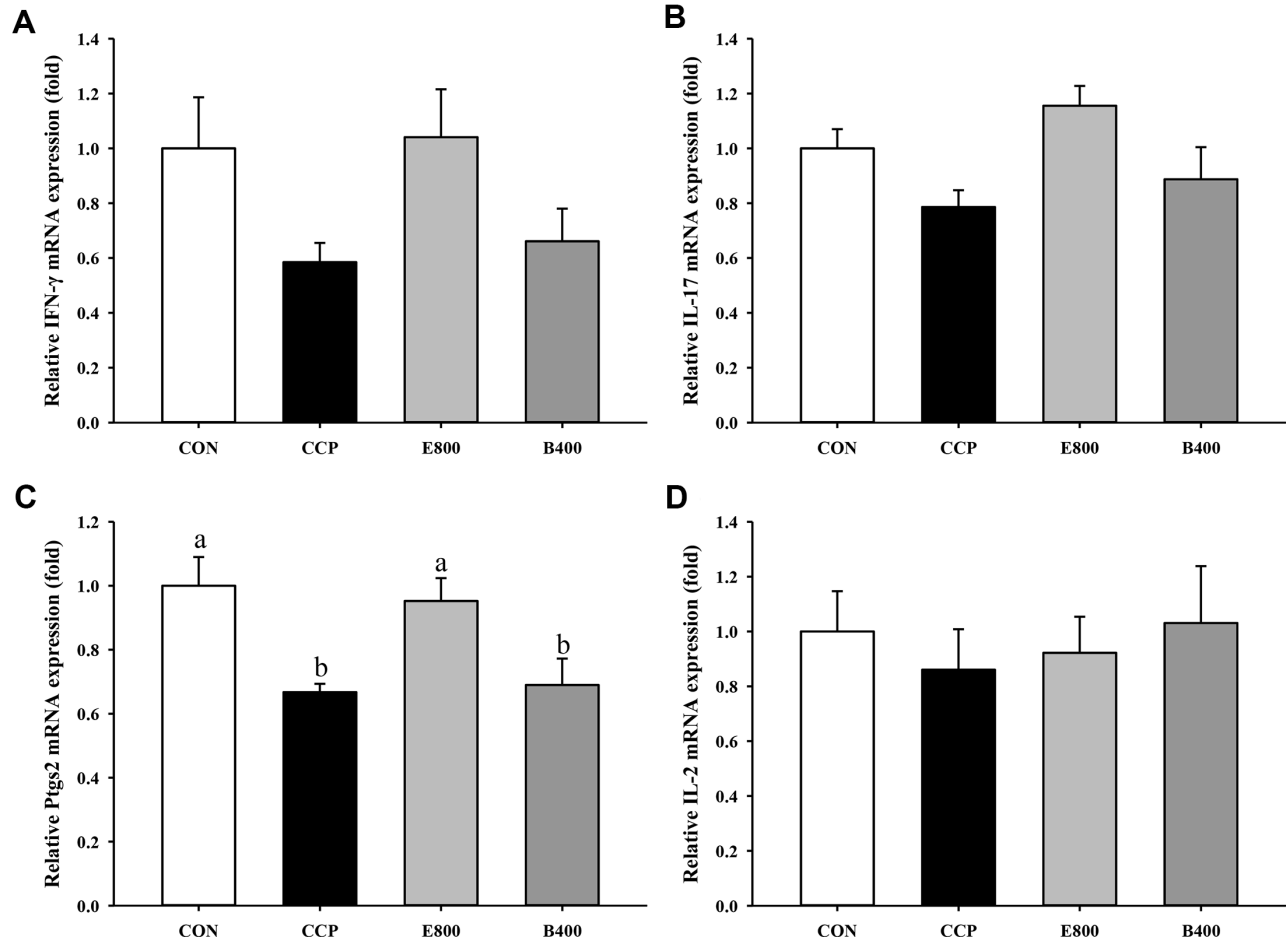
Effects of *Euglena* and β-glucan on the relative gene expression levels of cytokines and enzymes related to immunomodulation in CCP-induced immunosuppressed mice. CON, normal control; CCP, cyclophosphamide (80 mg/kg B.W.); E800, *Euglena* (800 mg/kg B.W.) and cyclophosphamide (80 mg/kg B.W.). B400, β-glucan (400 mg/kg B.W.) and cyclophosphamide (80 mg/kg B.W.) (*n* = 5, randomly selected mice for each group). Total RNA was extracted from the splenocytes of the model mice. The data represent the mean ± SEM. Different letters indicate significant differences (*p* < 0.05) according to Duncan’s multiple range test.

The most significantly regulated immune pathway in both the CCP vs. E800 and CCP vs. B400 comparisons was the IL-17 signaling pathway, and the most significantly modified DEG regulated by *Euglena* was Ptgs2. The expression levels of IL-17 pathway-related genes (IFN-γ, IL-17, and IL-2) and Ptgs2 were validated using RT‒qPCR (Fig. 6). The results revealed that, compared with those in the CON group, the expression levels of IFN-γ, IL-17, Ptgs2, and IL-2 in the CCP group decreased by 41.5%, 21.4%, 33.3%, and 14%, respectively. Notably, Ptgs2 gene expression in the E800 group was significantly greater than that in the CCP group (*p* = 0.006). The E800 group also exhibited approximately 77.9%, 46.9%, and 7.2% increases in the relative mRNA expression levels of IFN-γ, IL-17, and IL-2, respectively, compared to those in the CCP group, although these differences were not statistically significant. The expression levels of these genes tended to increase by 13.1%, 12.8%, and 19.8% in the B400 group compared to the CCP group.

## Discussion

*Euglena gracilis* contains a carbohydrate storage substance, β-glucan, which is a naturally occurring polysaccharide in the biological realm and exhibits various activities within the human body, including immune-enhancing effects [30]. Despite their recognized positive impact on the immune system, there remains a gap in direct research elucidating the genetic mechanisms involved in immune-regulatory effects. Therefore, in this study, we aimed to unravel these mechanisms through transcriptome analysis. However, it is difficult to confirm the immunomodulatory effect of a sample in healthy people or animals. The immunosuppressive function of CCP provides valuable insights into the challenging assessment of immune-related effects. Notably, research has highlighted enhancements in T and B lymphocyte proliferation and an increased count of immune cells following sample treatment in comparison to those in the CCP-induced mouse model [31]. In this study, significant decreases in the B.W. and weight of immune organs, including the spleen, Peyer's patches, and mesenteric lymph nodes (MLNs), after CCP treatment suggested an immunosuppressive effect of CCP. Immune activity occurs predominantly at key sites, such as the spleen, where immune functions are affected and potential damage occurs [32]. Specifically, the spleen, which includes numerous immune cells, is recognized for its role in diverse cytokine secretion pathways and the regulation of immune responses. Following the reduction in the spleen index induced by CCP, subsequent increases in the spleen index were observed in the E800 and B400 groups, signifying the immune-enhancing effects of *Euglena* and β-glucan. Additionally, splenocytes were extracted from the spleen and used for analyzing the transcriptome in the present study to confirm the importance of the modified immune system.

Transcriptome analysis is primarily utilized to determine the overall patterns of gene expression in cells or tissues, providing insights into biological processes and diseases [33]. The validated RNA sequencing results indicated that most of the immune-related pathways exhibited similar trends in the CON vs. E800 and CCP vs. B400 sets. However, compared with those in the β-glucan set, the DEG set in the *Euglena* cohort showed a more diverse and intricate array of DEGs and associated mechanisms.

The IL-17 signaling pathway was the most significantly modulated pathway in both the *Euglena*- and β-glucan-treated groups, as indicated by the KEGG database analysis. CD4+ T cells differentiate into various phenotypes, including Th1, Th2, and Th17 phenotypes [34]. IFN-γ produced by NK cells usually activates IL-17 receptors, contributing to the modulation of autoimmune diseases such as arthritis by inhibiting the differentiation of Th17 cells [34, 35]. Additionally, pathways associated with TNF and MAPK were significantly impacted by *Euglena* and β-glucan. Various immune receptors act through the MAPK signaling pathway to regulate the differentiation of cytokines and T cells [36]. These findings suggest that oral consumption of *Euglena* and β-glucan primarily influences T-cell differentiation and activation through the MAPK signaling pathway and concurrently impacts immune regulation through the secretion of cytokines such as IL-17 and TNF-α. GO analysis revealed that the genes related to the response to cytokines and lipopolysaccharides were specifically modulated by *Euglena*, which was not evident in the β-glucan DEG set. The diverse immune modulation effects of *Euglena*, surpassing those of β-glucan, may signify the immunomodulatory abilities of other constituents within *Euglena*, such as chlorophylls and carotenoids [37].

The functional connections among the 7 immune-related genes (Fosb, Fos, Jun, Cxcl2, Dusp1, Ptgs2, and Gabbr1) were confirmed by the PPI network and showed trends similar to but different from those of the *Euglena* and β-glucan DEG sets. Notably, Fosb, Fos, and Jun, which are closely related, encode the transcription factor activator protein-1 (AP-1), which plays crucial roles in activating T and B cells, differentiating T helper cells, and modulating cytokines through the MAPK signaling pathway [38]. Cxcl2, the gene most significantly regulated by β-glucan, is a chemokine produced by binding to G protein-coupled receptors found in immune cells [39]. This gene holds promise for offering valuable insights into the treatment of inflammatory and immune-related diseases, including rheumatoid arthritis [39].

Gabbr1 and Ptgs2 were exclusively identified in the *Euglena*-treated group but not in the β-glucan group. Gabbr1, which encodes the GABA receptor, regulates MAPK activity, influencing cytokine production and contributing to the reduction of inflammation [40]. GABA, which is secreted by NK cells, plays multifaceted roles in T-cell regulation, cytokine secretion, proliferation, and cytotoxicity [41]. Ptgs2, the key enzyme in prostaglandin biosynthesis, regulates inflammation and immune responses through Toll-like receptors and is induced by transcription factors such as NF-kB, MAPK, and JNK [42]. Ptgs2, also known as COX-2, primarily generates PGE2, promoting the differentiation of Th17 cells and exacerbating inflammation [43]. In one study, COX-2 was shown to interact more closely with other immune-related genes than Gabbr, and controlling Ptgs2 regulated by *Euglena* could lead to a potent anti-inflammatory effect [44]. *Euglena* synthesizes carbohydrate reserves utilizing the energy obtained through photosynthesis in its chloroplasts [45]. Carotenoids, which are synthesized in the chloroplasts and chromoplasts of microalgae and plants, are well known for their antioxidant effects and anti-inflammatory properties, as are chlorophyll. Carotenoids in *Euglena* promote T and B lymphocyte responses, enhance cytotoxic T cells and NK cell activity, and regulate COX-2 [21, 46, 47]. Additionally, chlorophyll components impact immunity, as indicated by increased IL-2 and IFN-γ levels within Peyer's patches and an increase in CD4+ T cells [48]. Previous research has shown that carotenoids extracted from *Euglena* exhibit anti-inflammatory effects by modulating the NF-kB signaling pathway [49]. It is reasonable to hypothesize that Gabbr1 and Ptgs2 are ultimately regulated by these pigment components in *Euglena*, extending beyond the influence of β-glucan. Further study of the beneficial components of *Euglena* could significantly contribute to elucidating the immune modulation mechanisms of *Euglena*.

In this study, we identified immune pathway and gene modifications in CCP-induced mice treated with *Euglena* and β-glucan, successfully constructing a network. Transcriptome profiling revealed increases in the expression levels of immune and inflammatory genes within the IL-17 and cAMP signaling pathways after treatment with *Euglena*. *Euglena* exhibited more diverse and abundant immune mechanisms than β-glucan, specifically involving two genes, Ptgs2 and Gabbr1, whose expression was suggested to be regulated by beneficial components such as carotenoids, which are known for enhancing the immune system. The transcriptome database can contribute to identifying key mechanisms influenced by immune modulation in *Euglena*. Further research into the positive effects of *Euglena*'s metabolically active compounds on immune responses is imperative to explore the benefits of these compounds as functional materials. Phenotypes of targeted immune diseases, such as rheumatoid arthritis, can be selected to contribute positively to therapeutic research. For accurate construction of the immune network and obtaining results, integrated data will be needed, utilizing not only RNA expression but also a broad range of biotechnological methods.

## Supplemental Materials

Supplementary data for this paper are available on-line only at http://jmb.or.kr.



## Figures and Tables

**Table 1:** Primers used for RT‒qPCR analysis.

Gene	Forward (5'-3')	Reverse (3'-5')
IFN-γ	atctggaggaactggcaaaa	ttcaagacttcaaagagtctgagg
IL-17	gctgacccctaagaaacccc	gaagcagtttgggacccctt
Ptgs2	gcccattgaacctggactg	acccaatcagcgtttctcgt
IL-2	gctgttgatggacctacagga	ttcaattctgtggcctgctt
GAPDH	aagagggatgctgcccttac	ccattttgtctacgggacga

TNF-α, tumor necrosis factor-alpha; IFN-γ, interferon-γ; IL-17, interleukin-17; Ptgs2, Cyclooxygenase-1; IL-2, interleukin-2; GAPDH, glyceraldehyde 3-phosphate dehydrogenase

**Table 2:** Effects of *Euglena* and β-glucan on the immune-related organ weight.

Group	Organ weight (g)		
	Spleen	Peyer’s patch	Mesenteric Lymph Node
CON	0.133 ± 0.004^a^	0.053 ± 0.003^a^	0.079 ± 0.001^a^
CCP	0.053 ± 0.003^d^	0.026 ± 0.002^b^	0.027 ± 0.002^b^
E800	0.080 ± 0.004^b^	0.032 ± 0.003^b^	0.027 ± 0.002^b^
B400	0.069 ± 0.002^c^	0.030 ± 0.002^b^	0.023 ± 0.001^b^

CON, normal control; CCP, cyclophosphamide (80 mg/kg B.W.); E800, *Euglena* (800 mg/kg B.W.) and cyclophosphamide (80 mg/kg B.W.); B400, β-glucan (400 mg/kg B.W.) and cyclophosphamide (80 mg/kg B.W.) (*n* = 13 for each group). The data represent the mean ± SEM. Different letters indicate significant differences (*p* < 0.05) according to Duncan’s multiple range test.

**Table 3:** Numbers of DEGs annotated to GO and KEGG.

DEG set	Filtered ID	Up-regulated gene	Down-regulated gene	Total gene (DEGs)	Total gene (GO)	Total gene (KEGG)
CON vs. CCP	16,315	1,383	929	2,312	1,922	905
CCP vs. E800	17,300	161	6	167	30	12
CCP vs. B400	16,998	30	2	32	15	10

CON, normal control; CCP, cyclophosphamide (80 mg/kg B.W.); E800, *Euglena* (800 mg/kg B.W.) and cyclophosphamide (80 mg/kg B.W.); B400, β-glucan (400 mg/kg B.W.) and cyclophosphamide (80 mg/kg B.W.) (*n* = 5, randomly selected mice for each group). Filtered ID, the number of samples with a read count > 2; Upregulated gene, the number of genes with a differential expression at FDR < 0.05 & log2 Fold Change ≥ 1; Downregulated gene, the number of genes with a differential expression at FDR < 0.05 & log2 Fold Change ≤ -1.

**Table 4:** Numbers of immune-related KEGG pathways and GO Biological Process (GO_BP) terms.

Method	DEG set	Pathway		Gene	
		Total	Immune-related	Total	Immune-related
KEGG	CCP vs. E800	16	5	10	7
	CCP vs. B400	11	3	6	5
GO_BP	CCP vs. E800	25	11	14	4
	CCP vs. B400	18	10	6	3

CON, normal control; CCP, cyclophosphamide (80 mg/kg B.W.); E800, *Euglena* (800 mg/kg B.W.) and cyclophosphamide (80 mg/kg B.W.). B400, β-glucan (400 mg/kg B.W.) and cyclophosphamide (80 mg/kg B.W.) (*n* = 5, randomly selected mice for each group). The ‘Gene’ category indicates the total number of genes annotated to the pathways and GO_BP.

**Table 5:** Immune-related KEGG pathways and GO Biological Process (GO_BP) terms after treatment with *Euglena*.

Type	Code	Term	P-value	Count	Gene
KEGG	mmu04657	IL-17 signaling pathway	3.32E-06	5	Fosb, Fos, Ptgs2, Jun, Cxcl2
KEGG	mmu04668	TNF signaling pathway	2.94E-04	4	Fos, Ptgs2, Jun, Cxcl2
KEGG	mmu04915	Estrogen signaling pathway	1.11E-02	3	Fos, Gabbr1, Jun
KEGG	mmu04024	cAMP signaling pathway	2.82E-02	3	Fos, Gabbr1, Jun
KEGG	mmu04010	MAPK signaling pathway	4.80E-02	3	Fos, Dusp1, Jun
GO_BP	GO:0051591	Response to cAMP	1.03E-03	3	Jun, Fos, Fosb
GO_BP	GO:0034097	Response to cytokine	2.90E-03	3	Jun, Fos, Ptgs2
GO_BP	GO:0009410	Response to xenobiotic stimulus	3.49E-03	4	Jun, Fos, Ptgs2, Fosb
GO_BP	GO:0071277	Cellular response to calcium ion	3.72E-03	3	Jun, Fos, Fosb
GO_BP	GO:0032496	Response to lipopolysaccharide	1.42E-02	3	Jun, Fos, Ptgs2
GO_BP	GO:0035994	Response to muscle stretch	1.87E-02	2	Jun, Fos
GO_BP	GO:0051412	Response to corticosterone	2.55E-02	2	Fos, Fosb
GO_BP	GO:0071276	Cellular response to cadmium ion	3.04E-02	2	Jun, Fos
GO_BP	GO:0032570	Response to progesterone	3.52E-02	2	Fos, Fosb
GO_BP	GO:0032870	Cellular response to hormone stimulus	4.00E-02	2	Fos, Fosb
GO_BP	GO:0034614	Cellular response to reactive oxygen species	4.19E-02	2	Jun, Fos

**Table 6:** Immune-related KEGG pathways and GO Biological Process (GO_BP) terms after treatment with β-glucan.

Type	Code	Term	P-value	Count	Gene
KEGG	mmu04657	IL-17 signaling pathway	8.55E-05	4	Fosb, Fos, Jun, Cxcl2
KEGG	mmu04668	TNF signaling pathway	5.30E-03	3	Fos, Jun, Cxcl2
KEGG	mmu04010	MAPK signaling pathway	3.28E-02	3	Fos, Dusp1, Jun
GO_BP	GO:0051591	Response to cAMP	4.98E-04	3	Fosb, Fos, Jun
GO_BP	GO:0071277	Cellular response to calcium ion	1.82E-03	3	Fosb, Fos, Jun
GO_BP	GO:0035994	Response to muscle stretch	1.31E-02	2	Fos, Jun
GO_BP	GO:0051412	Response to corticosterone	1.79E-02	2	Fosb, Fos
GO_BP	GO:0009410	Response to xenobiotic stimulus	1.94E-02	3	Fosb, Fos, Jun
GO_BP	GO:0071276	Cellular response to cadmium ion	2.14E-02	2	Fos, Jun
GO_BP	GO:0032570	Response to progesterone	2.48E-02	2	Fosb, Fos
GO_BP	GO:0032870	Cellular response to hormone stimulus	2.82E-02	2	Fosb, Fos
GO_BP	GO:0034614	Cellular response to reactive oxygen species	2.95E-02	2	Fos, Jun
GO_BP	GO:0009612	Response to mechanical stimulus	4.03E-02	2	Fosb, Jun
