# Supplementary material for: Unveiling Immunomodulatory Effects of Euglena gracilis in Immunosuppressed Mice: Transcriptome and Pathway Analysis
Source: J Microbiol Biotechnol. 2024 Feb 19;34(4):880–90. doi: 10.4014/jmb.2401.01006 (PMC11091698; doi:10.4014/jmb.2401.01006)
Supplement: Supplementary file 1 [file jmb-34-4-880-supple.pdf]

J. Microbiol. Biotechnol.
https://doi.org/10.4014/jmb.2401.01006

Supplemenary Material 1.

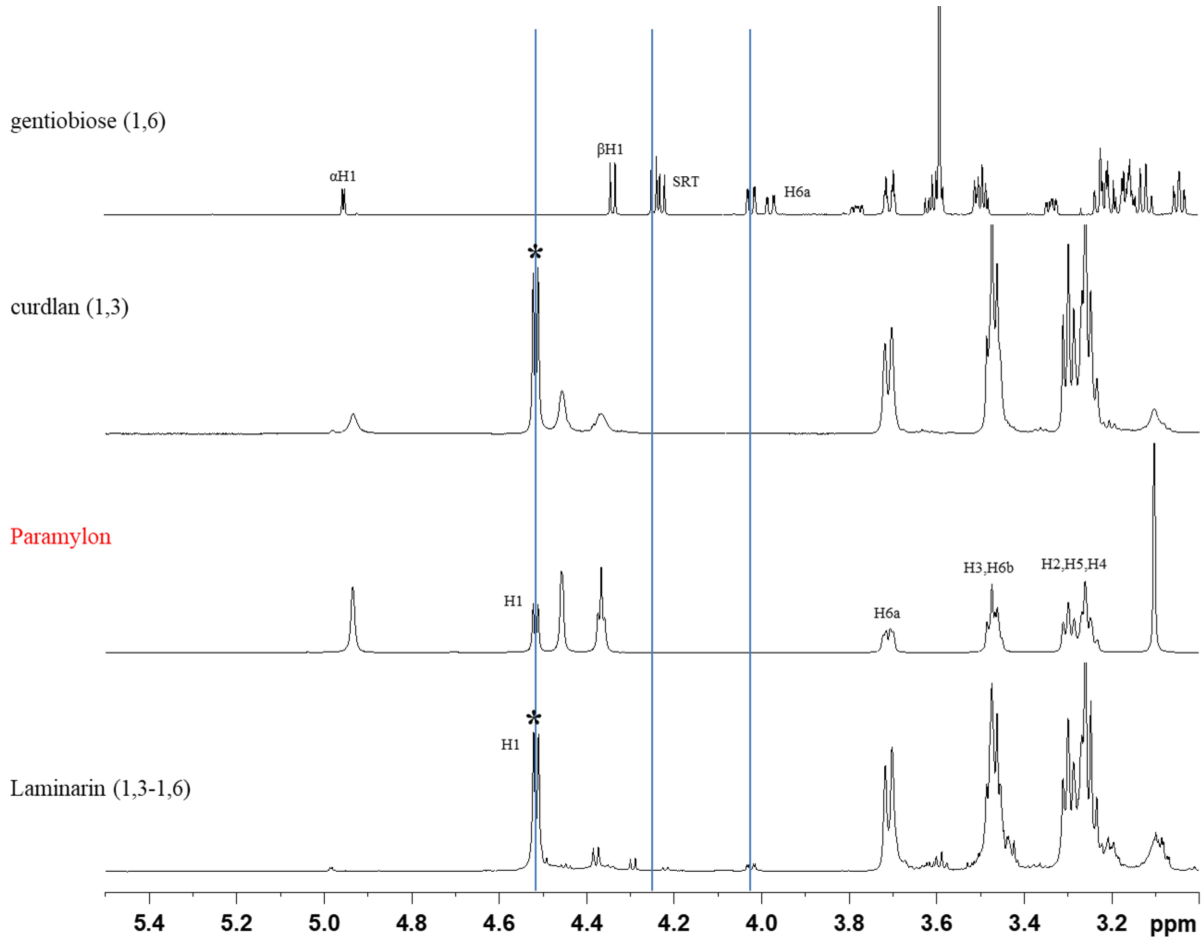

Confirmation of β-1,3-glucan through quantification analysis of β-glucans using NMR spectroscopy.
